# Association of the Platelets to High Density Lipoprotein Cholesterol Ratio and Risk of Heart Disease Events in Middle-Aged and Elderly Chinese Population: A Retrospective Cohort Study Utilizing the CHARLS Database

**DOI:** 10.31083/RCM26403

**Published:** 2025-02-18

**Authors:** Yating Huang, Xin Hou, Fang Lv, Zheng Gong

**Affiliations:** ^1^Department of Endocrinology, The Third Affiliated Hospital of Anhui Medical University (Hefei First People’s Hospital), 230022 Hefei, Anhui, China; ^2^Department of Cardiology, The Third Affiliated Hospital of Anhui Medical University (Hefei First People’s Hospital), 230022 Hefei, Anhui, China

**Keywords:** platelets to high density lipoprotein cholesterol ratio, heart disease events, CHARLS

## Abstract

**Background:**

The association between the platelet to high-density lipoprotein cholesterol ratio (PHR) and the risk of a heart disease event remains unclear. This study aims to determine whether the PHR can identify individuals at high risk for heart disease events, with a particular focus on middle-aged and elderly Chinese individuals.

**Methods:**

The retrospective cohort study encompassed 7188 middle-aged and elderly participants (>45 years) sourced from the China Health and Retirement Longitudinal Study (CHARLS) database. This research utilized longitudinal data from 5 follow-up visits spanning 2011 to 2020, which encompassed the collection of demographic profiles and pertinent blood biomarkers. Kaplan-Meier survival analysis was conducted based on PHR quartiles, with differences assessed using the log-rank test. The Cox proportional hazards model evaluated PHR’s hazard ratio (HR) as a predictor of outcome events, with trend tests applied. Restrictive cubic splines (RCS) were employed to explore associations. Subgroup analyses were performed to validate the robustness of the findings.

**Results:**

Baseline comparisons across quartiles of the PHR revealed a progressive increase in PHR values (133.16 vs 202.09 vs 267.04 vs 388.24), which corresponded to ascending incidence rates of heart disease (18.20% vs 18.64% vs 18.86% vs 21.59%) (*p < *0.05). The Kaplan-Meier survival analysis of PHR quartile groups revealed a notable elevation in the incidence of cardiovascular events in Q4 compared to Q1, Q2, and Q3 throughout the follow-up period (log-rank *p < *0.05). Upon adjustment for age, gender, stroke history, drinking, smoking, body mass index (BMI), white blood cell (WBC) count, fasting plasma glucose (FPG), creatinine (Cr), and triglyceride (TG), the Q4 group continued to exhibit a significantly elevated HR relative to Q1 (HR = 1.203, *p* = 0.023). Furthermore, RCS affirmed a linear association between PHR and heart disease events (Adjusted: Overall *p* = 0.014, Nonlinear *p* = 0.588). When analyzing by gender, high PHR was a risk factor for males (Q4: HR = 1.352, *p* = 0.019), but not for females (Q4: HR = 1.158, *p* = 0.166). Subgroup analysis indicates a significant association between higher PHR levels and increased risk of cardiac events compared to lower levels.

**Conclusions:**

Our study reveals a positive correlation between PHR levels and the incidence of heart disease events in middle-aged and elderly men in China. However, no such correlation was observed in female patients.

## 1. Introduction

Cardiovascular disease (CVD) currently accounts for approximately one-third of all global deaths, with a notable increase of 12.5% over the past decade [1], which was the primary cause of mortality among both urban and rural residents in China. The 2022 China Cardiovascular Health and Disease Report emphasizes the widespread impact of CVD, estimating 330 million cases. This includes 11.39 million cases of coronary heart disease (CHD), 8.9 million cases of heart failure, as well as significant numbers of other conditions such as pulmonary heart disease, atrial fibrillation, rheumatic heart disease, and congenital heart disease [2]. Risk factors such as smoking, sedentary lifestyle, and aging contribute to the increasing incidence and mortality of CVD in China. Projections based on a Maldivian computer simulation model suggest a more than 50% annual increase in CVD events in China from 2010 to 2030, underscoring the urgent need for effective preventive measures [3]. As global management of heart disease transitions to prevention, it is crucial to identify reliable indicators of high cardiovascular risk.

The platelet to high-density lipoprotein cholesterol ratio (PHR) has emerged as a promising biomarker for assessing inflammatory and lipid metabolic health, which is essential for maintaining the balance between clotting and anti-inflammatory processes in the bloodstream. Its effectiveness is primarily attributed to the significant roles of platelets and high-density lipoprotein cholesterol (HDL-C) in vascular health and the immune system. Excessive or aberrantly activated platelets may contribute to the development of atherosclerosis, thereby increasing the risk of thrombosis. HDL-C, often referred to as “good cholesterol”, facilitates the removal of excess cholesterol from arteries and possesses antioxidant and anti-inflammatory properties, thereby safeguarding arterial health. In contrast to isolated platelet counts or HDL-C levels, PHR offers a more comprehensive assessment of the balance between thrombus formation and arterial health by integrating data from both metrics. Notably, PHR not only reflects the anti-inflammatory and antioxidant capabilities of the vasculature but also serves as an indicator of systemic inflammatory responses. Under inflammatory conditions, platelet activation is enhanced, while HDL-C levels decrease. Consequently, PHR can act as a more sensitive marker for monitoring the progression of inflammatory diseases, such as atherosclerosis and myocarditis. Additionally, the total cholesterol-to-HDL-C ratio is commonly utilized for evaluating cardiovascular health; however, this approach overlooks the role of platelets. By incorporating both platelets and HDL-C, PHR provides a more comprehensive evaluation of potential thrombotic risk. Preliminary research suggests that elevated PHR levels may correlate with an increased risk of atherosclerosis, thrombosis, and metabolic syndrome [4]. Furthermore, PHR has shown promise in predicting the severity of coronary artery stenosis and the prognosis of patients with CHD [5, 6]. Nevertheless, the capacity of PHR to effectively predict future heart disease risk remains uncertain and requires further investigation [7].

Currently, there is a lack of studies exploring the predictive value of PHR specifically for heart disease occurrence in middle-aged and elderly populations. This study aims to address this gap using data from the CHARLS database, focusing on middle-aged and elderly Chinese individuals. Its objective is to determine whether PHR can identify high-risk individuals for heart disease events, potentially informing targeted preventive strategies in these high-risk groups.

## 2. Methods and Materials 

### 2.1 Study Population

The China Health and Retirement Longitudinal Study (CHARLS) is a comprehensive national longitudinal social survey database encompassing middle-aged and elderly individuals in China [8]. Initiated in 2011, CHARLS is a collaborative effort between the Institute of Economics at the Chinese Academy of Social Sciences and the China Center for Economic Research at Peking University, with funding from the National Institutes of Health (NIH), the National Science Foundation (NSF), among others. This database integrates various data modalities, including structured questionnaires, physiological measurements, and biological samples, spanning urban and rural regions nationwide, and ensuring national representativeness.

**Fig. 1.**
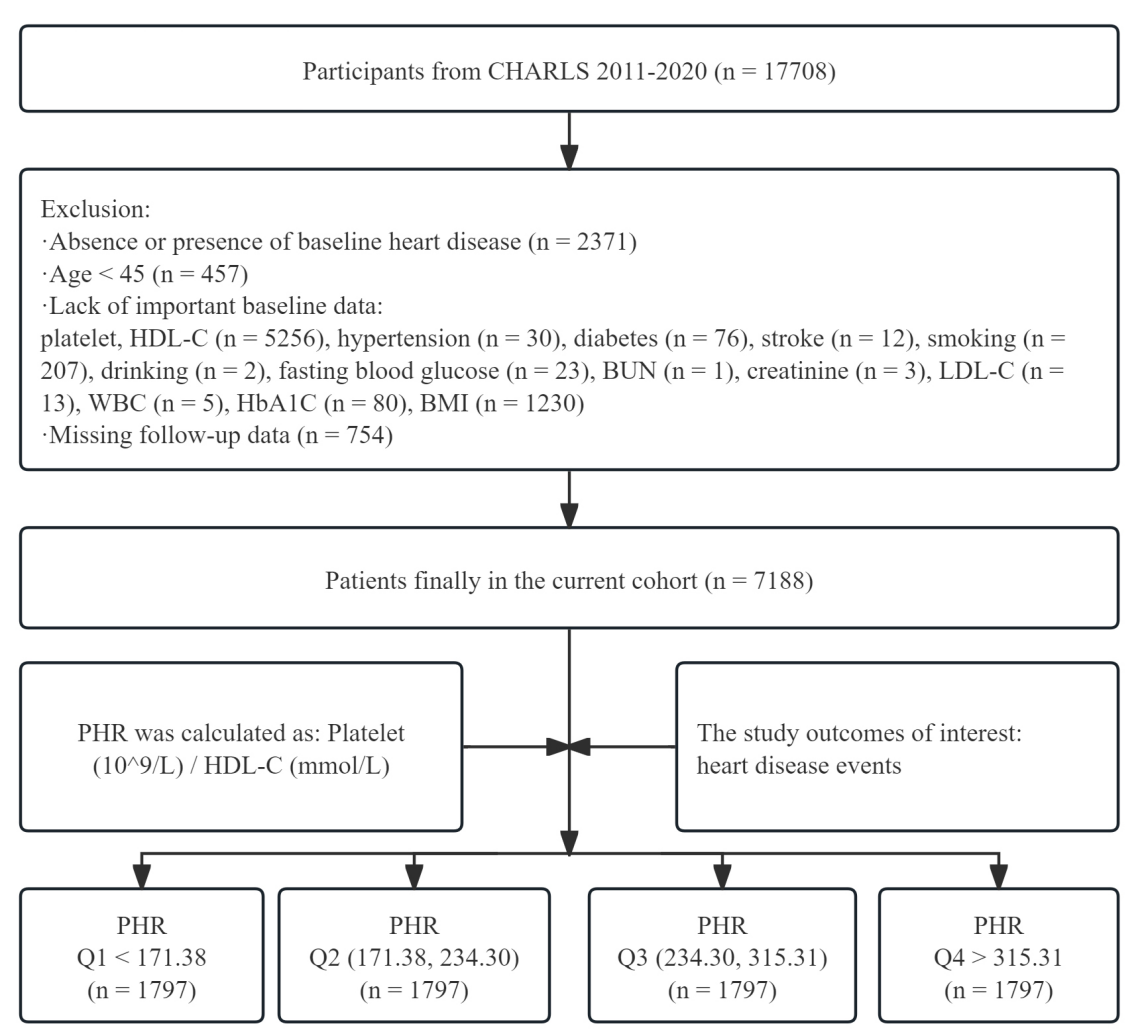
**Flow chart for inclusion of participants**. CHARLS, China Health and Retirement Longitudinal Study; HDL-C, high density lipoprotein cholesterol; BUN, blood urea nitrogen; LDL-C, low density lipoprotein cholesterol; HbA1C, glycosylated hemoglobin; BMI, body mass index; PHR, platelets to high density lipoprotein cholesterol ratio; WBC, white blood cell; Q1, quartile 1 of PHR; Q2, quartile 2 of PHR; Q3, quartile 3 of PHR; Q4, quartile 4 of PHR.

This research utilized longitudinal data from 5 follow-up visits spanning 2011 to 2020, capturing demographic profiles and pertinent blood biomarkers. Exclusion criteria were applied to remove entries lacking essential baseline indicators or definitive heart disease status. The retrospective cohort study encompassed 7188 middle-aged and elderly participants (>45 years) sourced from the CHARLS database (Fig. 1).

### 2.2 Definition of Exposure Variables and Outcome Events

In this study, PHR was defined as the ratio of platelet count (×10^12^/L) to HDL-C (mmol/L) [4, 5, 7]. Collection of test indicators: after fasting for at least 8 hours in the morning, participants provide blood samples at designated medical institutions or laboratories. The laboratory records the results in a database. Following standard operational protocols and regular data audits to ensure accuracy and reliability, the results are then included in the CHARLS database for analysis and research by researchers and policymakers. Heart disease, as defined for this study, encompassed conditions such as coronary artery disease, angina, heart failure, and other cardiac disorders, including those diagnosed by a healthcare provider. During follow-up assessments, participants were asked the question: “Have you ever received a diagnosis of heart disease, such as coronary artery disease, angina, congestive heart failure, or another heart condition from a healthcare professional”? Incidence of heart disease events was documented for participants reporting at least one heart attack.

### 2.3 Statistical Analyses

This study utilized data was analyzed with the use of the statistical packages R (The R Foundation; http://www.r-project.org; version 4.2.0) and EmpowerStats (http://www.empowerstats.net/, X&Y solutions, Inc. Boston, MA, USA) for statistical analyses. The study cohort was stratified into four groups based on quartiles of the PHR: Q1 (n = 1797, PHR <171.38), Q2 (n = 1797, 171.38 ≤ PHR < 234.30), Q3 (n = 1797, 234.30 ≤ PHR < 315.31), and Q4 (n = 1797, PHR ≥315.31). Descriptive statistics are presented as mean ± standard deviation for normally distributed data and as the median (P25, P75) for skewed distributions. Analytical methods appropriate to data distribution—such as the Kruskal-Wallis test, analysis of variance, and Chi-square test—were selected accordingly. Kaplan-Meier survival analysis was conducted based on PHR quartiles, with differences assessed using the log-rank test. The Cox proportional hazards model evaluated the HR of PHR as a predictor of outcome events, with trend tests applied. Restrictive cubic spline (RCS) was employed to explore associations. Subgroup analyses were performed to validate the robustness of the findings. A significance threshold of bilateral *p* < 0.05 was applied throughout the analyses.

## 3. Results 

### 3.1 Characteristics of the Study Population Based on PHR Quartiles

In this study, a total of 7188 subjects were enrolled, with a median follow-up period of 108 months. Among them, 1389 participants (19.32%) experienced a cardiovascular event. Baseline comparisons across quartiles of the PHR revealed a progressive increase in PHR values (133.16 vs 202.09 vs 267.04 vs 388.24) corresponding to ascending incidence rates of heart disease (18.20% vs 18.64% vs 18.86% vs 21.59%) (Table 1). Significant differences (*p* < 0.05) were observed in age, white blood cell (WBC), platelet (PLT), uric acid (UA), body mass index (BMI), triglycerides (TG), HDL-C, low-density lipoprotein cholesterol (LDL-C), glycosylated hemoglobin (HbA1C), fasting plasma glucose (FPG), C-reactive protein (CRP), PHR, gender, drinking, hypertension, and diabetes mellitus (DM) (Table 1).

**Table 1.** **Baseline characteristics stratified by PHR levels**.

Variables		Total (n = 7188)	Q1 (<171.38)	Q2 (171.38, 234.30)	Q3 (234.30, 315.31)	Q4 (>315.31)	*p*
			(n = 1797)	(n = 1797)	(n = 1797)	(n = 1797)	
Age (years), Mean ± SD		58.77 ± 9.46	60.11 ± 9.82	59.19 ± 9.42	58.07 ± 9.23	57.69 ± 9.18	<0.001
Time (month), M (Q1, Q3)		108.00 (84.00, 108.00)	108.00 (84.00, 108.00)	108.00 (84.00, 108.00)	108.00 (84.00, 108.00)	108.00 (84.00, 108.00)	0.122
WBC (×10^9^/L), Mean ± SD		6.25 ± 1.90	5.69 ± 1.80	6.06 ± 1.66	6.41 ± 1.93	6.83 ± 1.99	<0.001
PLT (×10^9^/L), M (Q1, Q3)		207.00 (162.00, 255.00)	139.00 (111.00, 170.00)	192.00 (165.00, 222.00)	226.00 (197.00, 260.00)	273.00 (237.00, 311.00)	<0.001
Cr (mg/dL), Mean ± SD		0.78 ± 0.23	0.79 ± 0.31	0.77 ± 0.19	0.77 ± 0.19	0.77 ± 0.20	0.209
UA (mg/dL), Mean ± SD		4.41 ± 1.24	4.41 ± 1.20	4.35 ± 1.22	4.41 ± 1.24	4.48 ± 1.30	0.016
BMI (kg/m^2^), Mean ± SD		23.45 ± 3.86	22.51 ± 3.74	22.88 ± 3.64	23.80 ± 3.82	24.62 ± 3.87	<0.001
TC (mmol/L), Mean ± SD		3.39 ± 0.66	3.41 ± 0.63	3.40 ± 0.65	3.40 ± 0.67	3.36 ± 0.70	0.119
TG (mmol/L), Mean ± SD		2.22 ± 1.60	1.67 ± 0.92	1.94 ± 1.23	2.18 ± 1.30	3.08 ± 2.26	<0.001
HDL-C (mmol/L), Mean ± SD		0.90 ± 0.26	1.12 ± 0.28	0.96 ± 0.21	0.85 ± 0.17	0.68 ± 0.16	<0.001
LDL-C (mmol/L), Mean ± SD		2.05 ± 0.61	1.99 ± 0.56	2.07 ± 0.58	2.12 ± 0.60	2.02 ± 0.67	<0.001
HbAlC (%), Mean ± SD		5.27 ± 0.79	5.19 ± 0.67	5.24 ± 0.73	5.30 ± 0.83	5.34 ± 0.91	<0.001
FPG (mmol/L), M (Q1, Q3)		5.67 (5.24, 6.22)	5.61 (5.21, 6.08)	5.64 (5.25, 6.16)	5.65 (5.22, 6.23)	5.78 (5.30, 6.45)	<0.001
CRP (mg/L), M (Q1, Q3)		0.98 (0.54, 2.08)	0.82 (0.48, 1.73)	0.87 (0.49, 1.86)	1.01 (0.54, 2.10)	1.30 (0.70, 2.63)	<0.001
PHR, M (Q1, Q3)		234.30 (171.38, 315.31)	133.16 (109.39, 154.15)	202.09 (186.64, 218.79)	267.04 (250.04, 289.34)	388.24 (346.01, 461.48)	<0.001
Heart event, n (%)							0.043
	No	5799 (80.68)	1470 (81.80)	1462 (81.36)	1458 (81.14)	1409 (78.41)	
	Yes	1389 (19.32)	327 (18.20)	335 (18.64)	339 (18.86)	388 (21.59)	
Stroke, n (%)							0.081
	No	7037 (97.90)	1757 (97.77)	1771 (98.55)	1760 (97.94)	1749 (97.33)	
	Yes	151 (2.10)	40 (2.23)	26 (1.45)	37 (2.06)	48 (2.67)	
Gender, n (%)							0.027
	Female	3859 (53.69)	917 (51.03)	969 (53.92)	966 (53.76)	1007 (56.04)	
	Male	3329 (46.31)	880 (48.97)	828 (46.08)	831 (46.24)	790 (43.96)	
Drinking, n (%)							<0.001
	No	4754 (66.14)	1094 (60.88)	1150 (64.00)	1215 (67.61)	1295 (72.06)	
	Yes	2434 (33.86)	703 (39.12)	647 (36.00)	582 (32.39)	502 (27.94)	
Smoking, n (%)							0.093
	No	5009 (69.69)	1217 (67.72)	1248 (69.45)	1258 (70.01)	1286 (71.56)	
	Yes	2179 (30.31)	580 (32.28)	549 (30.55)	539 (29.99)	511 (28.44)	
Hypertension, n (%)							<0.001
	No	3903 (54.30)	1010 (56.20)	1026 (57.10)	968 (53.87)	899 (50.03)	
	Yes	3285 (45.70)	787 (43.80)	771 (42.90)	829 (46.13)	898 (49.97)	
Diabetes, n (%)							<0.001
	No	6135 (85.35)	1595 (88.76)	1556 (86.59)	1528 (85.03)	1456 (81.02)	
	Yes	1053 (14.65)	202 (11.24)	241 (13.41)	269 (14.97)	341 (18.98)	

SD, standard deviation; M, median; PHR, platelets to high density lipoprotein cholesterol ratio; WBC, white blood cell; PLT, platelet; Cr, creatinine; UA, uric acid; BMI, body mass index; TC, total cholesterol; TG, triglyceride; HDL-C, high density lipoprotein cholesterol; LDL-C, low density lipoprotein cholesterol; HbA1C, glycosylated hemoglobin; FPG, fasting plasma glucose; CRP, C-reactive protein; Q1, quartile 1 of PHR; Q2, quartile 2 of PHR; Q3, quartile 3 of PHR; Q4, quartile 4 of PHR.

### 3.2 Survival Analysis Based on PHR Quartiles

**Fig. 2.**
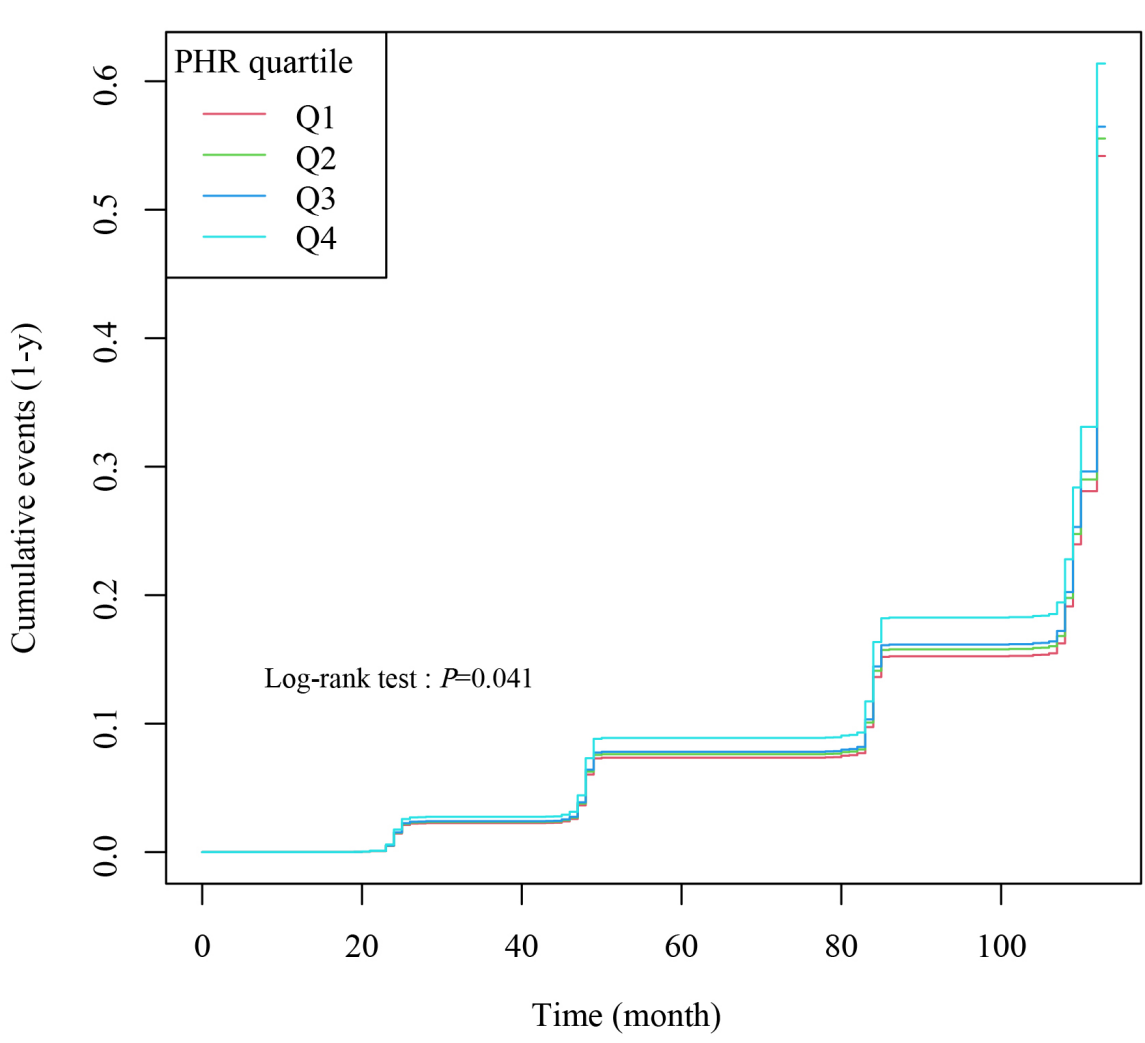
**Kaplan-Meier cumulative risk curve for cardiac events**. PHR, platelets to high density lipoprotein cholesterol ratio; Q1, quartile 1 of PHR; Q2, quartile 2 of PHR; Q3, quartile 3 of PHR; Q4, quartile 4 of PHR.

Based on the Kaplan-Meier survival analysis of PHR quartile groups, the incidence of cardiovascular events was notably elevated in Q4 compared to Q1, Q2, and Q3 throughout the follow-up period (log-rank *p* < 0.05) (Fig. 2). These findings indicate an increased risk of cardiovascular disease among individuals with higher PHR values.

### 3.3 Correlation of the PHR with Outcome Events

Using group Q1 as the reference, a Cox proportional hazards model was employed to assess the association between PHR and incidents of heart disease. Initially, without adjusting for pertinent risk factors, the hazard ratio (HR) in the Q4 group significantly exceeded that of Q1, underscoring a notable association between elevated PHR and heightened susceptibility to heart disease events (Q4: HR = 1.214, *p* = 0.009) (Table 2). Upon adjustment for age, gender, stroke history, drinking, smoking, BMI, WBC, FPG Cr, and TG, the Q4 group continued to exhibit a significantly elevated HR relative to Q1 (Q4: HR = 1.203, *p* = 0.023) (Table 2). When treating PHR as a continuous variable, each interquartile range increase was associated with a 4.4% rise in the risk of new heart events (HR = 1.044, *p* < 0.001) (Table 2). Additionally, trend testing confirmed a consistent escalation in heart disease risk with rising PHR values, irrespective of adjustment for confounders (Non-adjusted HR = 1.064, *p* = 0.010; Adjusted II HR = 1.058, *p* = 0.032) (Table 2). Finally, considering that the association between PHR and the occurrence of cardiovascular events might vary by gender, we performed separate proportional hazards regression analyses for men and women. The results indicate that high PHR is a significant risk factor for male (Q4: HR = 1.352, *p* = 0.019), but it may not be a relevant risk factor for female (Q4: HR = 1.158, *p* = 0.166).

**Table 2.** **Multivariate Cox regression analysis of PHR and risk of heart disease events**.

Exposure		Non-adjusted		Adjust I		Adjust II	
		HR (95% CI)	*p*	HR (95% CI)	*p*	HR (95% CI)	*p*
PHR		1.041 (1.019, 1.064)	<0.001	1.049 (1.027, 1.072)	<0.001	1.044 (1.019, 1.070)	<0.001
PHR quartile							
	Q1	1.0		1.0		1.0	
	Q2	1.036 (0.889, 1.206)	0.652	1.048 (0.900, 1.221)	0.545	1.051 (0.902, 1.226)	0.522
	Q3	1.056 (0.907, 1.230)	0.481	1.103 (0.947, 1.284)	0.208	1.056 (0.904, 1.235)	0.490
	Q4	1.214 (1.048, 1.407)	0.009	1.276 (1.101, 1.479)	0.001	1.203 (1.025, 1.412)	0.023
PHR for trend		1.064 (1.015, 1.115)	0.010	1.083 (1.033, 1.135)	0.001	1.058 (1.005, 1.114)	0.032
Male							
	Q1	1.0		1.0		1.0	
	Q2	0.982 (0.767, 1.258)	0.886	1.033 (0.806, 1.323)	0.800	1.052 (0.820, 1.350)	0.691
	Q3	1.028 (0.805, 1.314)	0.825	1.100 (0.860, 1.406)	0.449	1.076 (0.838, 1.381)	0.566
	Q4	1.305 (1.035, 1.646)	0.025	1.434 (1.135, 1.812)	0.003	1.352 (1.050, 1.742)	0.019
Female							
	Q1	1.0		1.0		1.0	
	Q2	1.103 (0.909, 1.337)	0.321	1.108 (0.914 1.344)	0.298	1.087 (0.895, 1.320)	0.399
	Q3	1.044 (0.858, 1.269)	0.669	1.086 (0.893 1.321)	0.408	1.022 (0.836, 1.250)	0.834
	Q4	1.186 (0.981, 1.434)	0.078	1.243 (1.028 1.504)	0.065	1.158 (0.941, 1.426)	0.166

Non-adjusted model adjust for: None. Adjust I model adjust for: Age; Gender. Adjust II model adjust for: Age; Gender; Stroke; Drinking; Smoking; BMI; WBC; FPG; Cr; TG; LDL-C; CRP; HbA1C; UA; Hypertension; Diabetes. PHR for trend: trend test of PHR and cardiac events. PHR, platelets to high density lipoprotein cholesterol ratio; HR, hazard ratio; CI, confidence interval; BMI, body mass index; WBC, white blood cell; FPG, fasting plasma glucose; Cr, creatinine; TG, triglyceride; LDL-C, low density lipoprotein cholesterol; CRP, C-reactive protein; HbA1C, glycosylated hemoglobin; UA, uric acid; Q1, quartile 1 of PHR; Q2, quartile 2 of PHR; Q3, quartile 3 of PHR; Q4, quartile 4 of PHR.

**Fig. 3.**
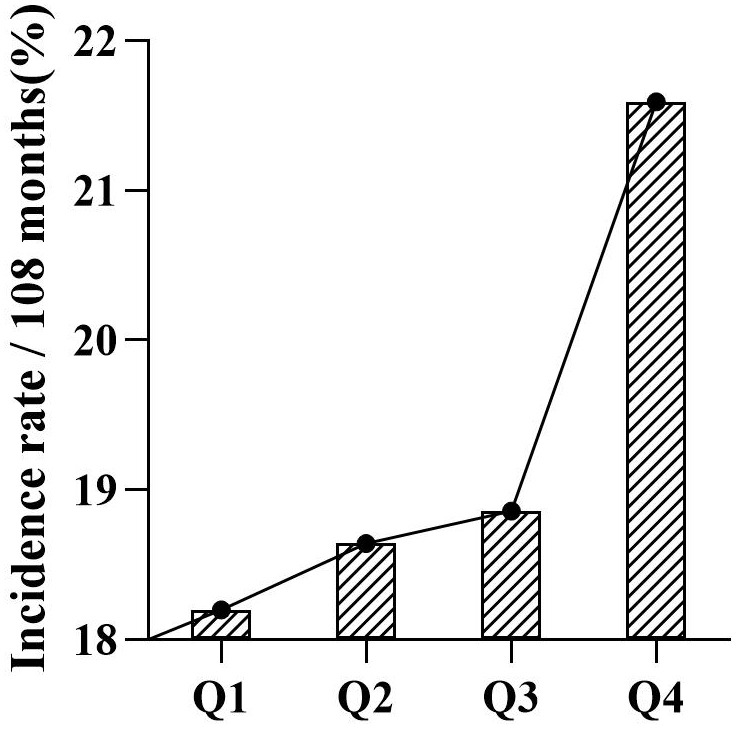
**Comparison of the incidence of cardiac events in the PHR quartile population**. PHR, platelets to high density lipoprotein cholesterol ratio.

**Fig. 4.**
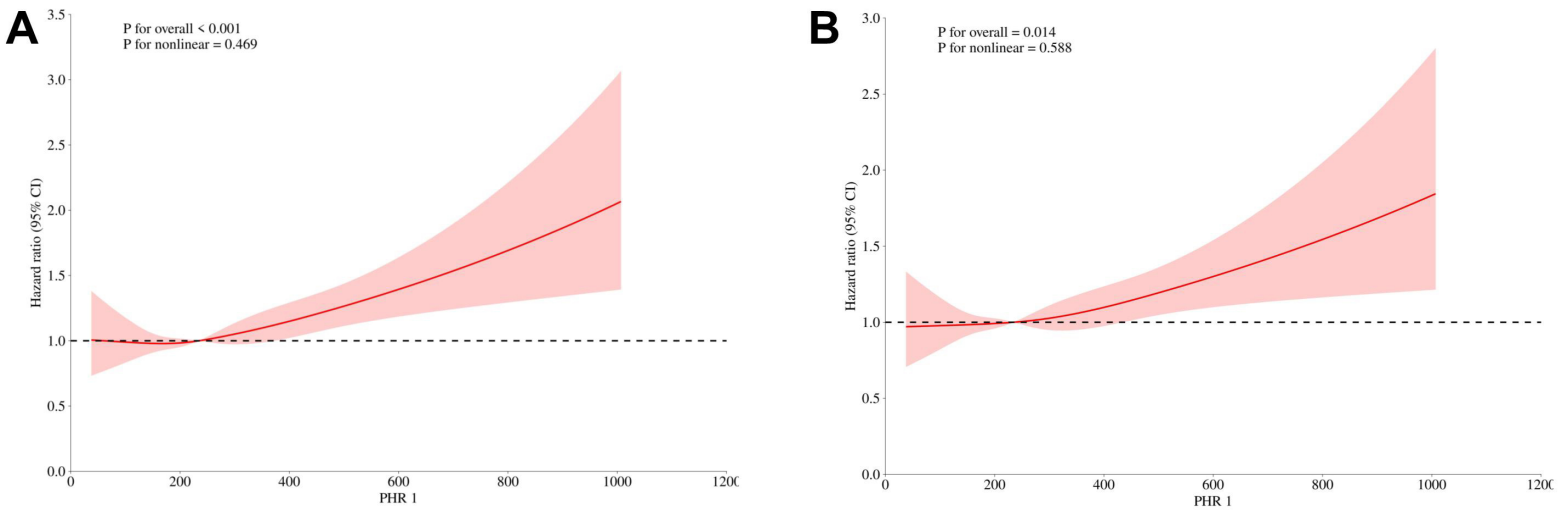
**Restricted cubic spline curve for PHR hazard ratio (A: Non-adjusted. B: Adjusted for: Age; Gender; Stroke; Drinking; Smoking; Hypertension; Diabetes)**. CI, confidence interval; PHR, platelets to high density lipoprotein cholesterol ratio.

To evaluate the linearity of the relationship between PHR and heart disease events, a bar chart was constructed. This graphical representation illustrates a progressive increase in heart disease events as PHR values ascend from Q1 to Q4 (Fig. 3). Furthermore, RCS analysis affirmed a linear association between PHR and heart disease events, maintaining significance across adjusted and non-adjusted models (Non-adjusted: Overall *p* < 0.001, Nonlinear *p* = 0.469; Adjusted: Overall *p* = 0.014, Nonlinear *p* = 0.588) (Fig. 4).

### 3.4 Subgroup Analysis

**Fig. 5.**
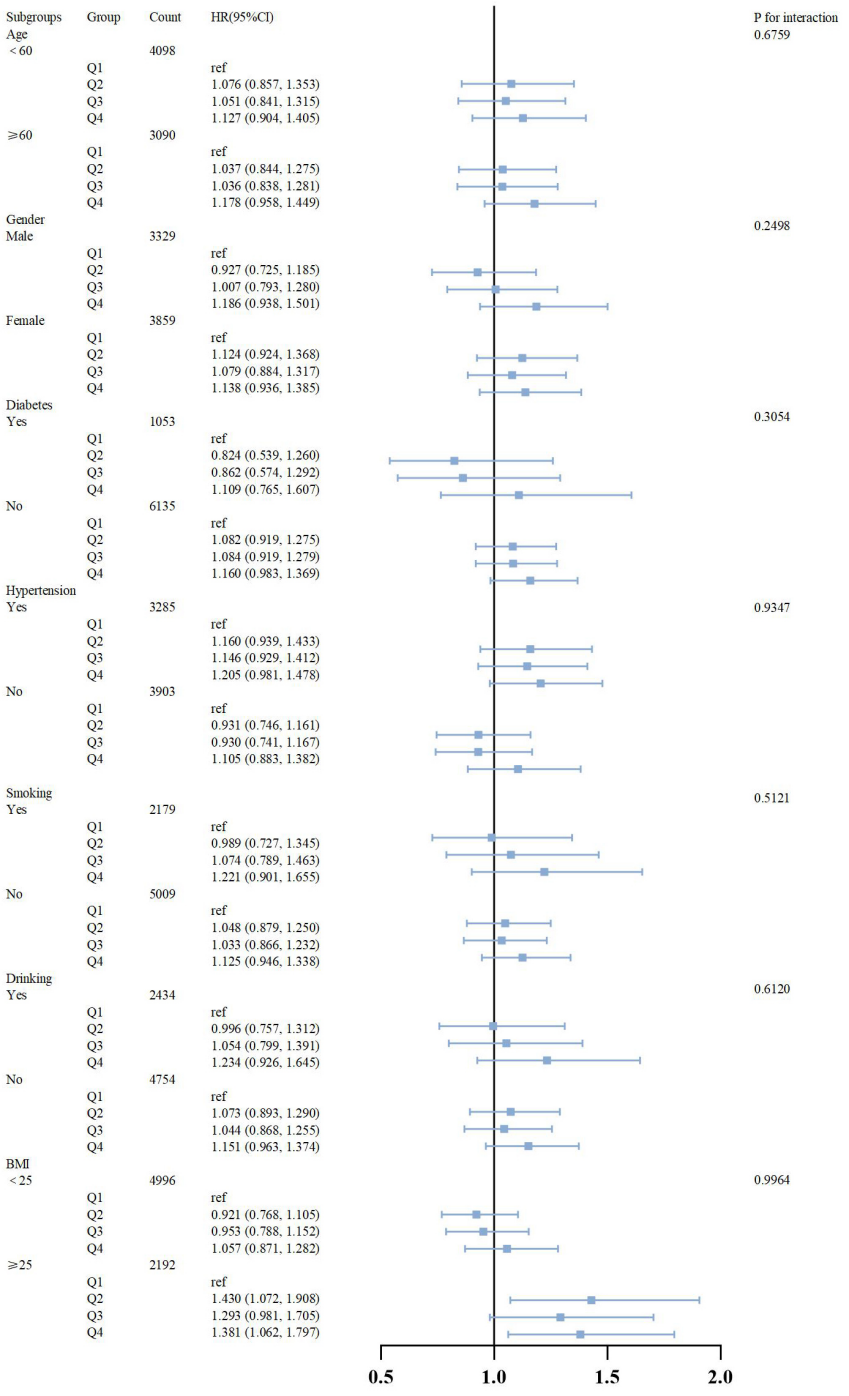
**Subgroup analysis of outcomes from cardiac events**. HR, hazard ratio; CI, confidence interval; BMI, body mass index; Q1, quartile 1 of PHR; Q2, quartile 2 of PHR; Q3, quartile 3 of PHR; Q4, quartile 4 of PHR.

To assess the robustness of the association between elevated PHR and the incidence of heart disease events, and to examine potential subgroup variations in this association, we categorized PHR into quartiles and stratified by common risk factors: age (<60 years vs ≥60 years), sex (male vs female), diabetes status (yes vs no), hypertension (yes vs no), smoking status (yes vs no), alcohol consumption (yes vs no), and BMI (<25 vs ≥25). The correlation between PHR and heart disease events was analyzed within each subgroup. Our findings indicate a significant association between higher PHR levels (Q4) and increased risk of cardiac events compared to lower levels (Q1) (Fig. 5). Furthermore, interaction testing revealed no significant interactions between PHR and any of the stratified variables (*p* > 0.05) (Fig. 5).

## 4. Discussion

Previous studies have shown associations between PHR and patient outcomes in metabolic syndrome, stroke, and cardiovascular disease [4, 5, 6]. However, the specific relationship between PHR and heart disease requires further elucidation. This retrospective cohort study analyzed data from 7188 participants in the CHARLS database spanning 2011 to 2020, with a median follow-up of 108 months. Controlling for pertinent confounders, our findings indicate a significant association between PHR and incident cardiac events. Furthermore, employing RCS regression revealed a linear trend, underscoring an incremental risk of heart disease events with higher PHR values. These results suggest that PHR holds promise as a prognostic indicator for heart disease occurrence.

PHR has been demonstrated to possess both inflammatory and lipid metabolic properties. Consequently, it is currently regarded as a reliable marker of inflammation. Previous research has extensively explored the relationship between PHR and lipid metabolic disorders as well as CVDs. A cohort study utilizing the 2005–2018 the National Health and Nutrition Examination Survey (NHANES) data has revealed a correlation between PHR and CVD mortality risk in stroke survivors [9]. However, this study was limited in scope, as it was conducted exclusively with stroke survivors, which limits the extent to which its findings can be generalized. This study builds upon previous research by examining the relationship between PHR and cardiac disease, with a particular focus on middle-aged and older populations, who are at elevated risk for cardiac disease and may potentially benefit from this investigation in a significant clinical manner. Moreover, a real-world study of coronary artery disease patients with type 2 diabetes mellitus (T2DM) revealed an association between PHR and long-term adverse outcomes [5]. This study’s narrow focus on coronary artery disease limits its applicability and primarily examines prognosis, whereas our research emphasizes the preventive aspects of PHR in cardiac disease. Furthermore, a study of patients with depression indicated a potential correlation between PHR and cardiovascular mortality in this population [10]. However, the focus of this study on prognosis contrasts with our focus on the risk of incident disease. Previous research has also investigated PHR in relation to metabolic syndrome risk factors such as hypertension, diabetes, and obesity, all of which are known risk factors for cardiac events. Nonetheless, direct associations between PHR and cardiac events have not been reported [4]. Furthermore, our study reveals that PHR shows a stronger association with cardiac events in men, whereas no significant correlation was observed in females. This could potentially be due to the higher estrogen levels in women, which reduce platelet activity compared to males.

Platelets, which originate from megakaryocytes, serve as crucial participants in hemostasis, thrombosis, and various physiological processes. They are increasingly being recognized as non-traditional risk factors in the context of CVD [7]. Recent studies indicate that platelets influence the development of CVD by modulating the properties of vascular endothelial cells, thereby promoting monocyte and lymphocyte infiltration into artery walls [11, 12, 13, 14, 15]. This platelet-mediated influence on endothelial cells is tightly regulated by receptors such as αIIbβ3 and αVβ3. This results in the activation of platelets and the subsequent release of pro-inflammatory and pro-atherogenic cytokines and chemokines [16, 17, 18, 19]. These factors adhere to endothelial surfaces, facilitating the recruitment of inflammatory cells like T cells. This, in turn, drives subsequent atherosclerosis and thrombosis, which are key contributors to CVD pathogenesis [20, 21, 22]. Additionally, elevated phospholipid levels in platelets among elderly individuals with CVD can result in the production of reactive oxygen species (ROS), which contribute to oxidative stress and are implicated in the pathophysiology of CVD. Moreover, a bivariate Mendelian randomization study incorporating data from UK Biobank (n = 350,475) and the International Consortium of Blood Pressure (ICBP) (n = 299,024) demonstrates a positive correlation between platelet counts and blood pressure, indicating that platelets may exacerbate CVD risk factors like hypertension [23]. These insights underscore the multifaceted role of platelets in cardiovascular health, highlighting potential avenues for therapeutic intervention in the prevention and management of CVD.

Numerous epidemiological studies have consistently identified low HDL-C as a significant risk factor for CVD, particularly in patients with early-onset coronary artery disease. The incidence of CVD is inversely proportional to HDL-C concentration [24]. Each 1 mg/dL increase in HDL-C is associated with a 2% to 3% lower risk of CVD [25]. HDL-C plays a crucial role in the reduction of atherosclerosis through cholesterol reverse transport, whereby HDL-C particles act as vehicles for the removal of cholesterol from peripheral tissues and its subsequent excretion in the liver [26]. The precise mechanisms underlying HDL-C’s regulatory and protective effects on endothelial function remain incompletely understood. Recent research has demonstrated that reconstituted HDL-C (rHDL-C) particles containing apolipoprotein A-I and phospholipids stimulate nitric oxide (NO) production in endothelial cells, inhibit apoptosis, and promote endothelial cell migration and re-endothelialization [27, 28, 29].

HDL-C and platelets engage in complex interactions that collectively regulate the onset and progression of CVD. HDL-C has been identified as an independent predictor of acute platelet thrombosis. It exerts antiplatelet effects by inhibiting processes such as platelet aggregation, fibrinogen binding, granule secretion, and thromboxane A2 release [30, 31]. Furthermore, HDL-C facilitates vascular dilation, enhancing blood flow and restraining platelet inflammatory activation and thrombosis through endothelial cell signaling pathways. Activation of protein kinase B (PKB), mitogen-activated protein kinase (p42/44MAPK), Ca^2+^ calmodulin-dependent protein kinase, and adenosine monophosphate (AMP)-activated protein kinase (AMPK) collectively promote the release of vasodilator factors and the production of endothelial NO, thereby further inhibiting platelet activation, atherosclerosis, and thromboinflammatory responses [32, 33, 34, 35, 36]. Additionally, HDL-C enhances prostacyclin (PGI2) synthesis and acts in conjunction with NO to inhibit platelet activation, thus reducing thrombotic risk and the incidence of CVD [37, 38, 39, 40, 41]. Conversely, platelets facilitate the formation of foam cells by enhancing cholesterol ester formation and accumulation in monocyte-derived macrophages which are circulating in the periphery, thereby promoting atherosclerosis and elevating the risk of CVD [42, 43].

PHR emerges as a composite marker reflecting both inflammation and lipid metabolism, offering a straightforward, cost-effective assessment readily applicable in clinical settings. A substantial body of research has underscored the correlation between PHR and cardiovascular diseases. For instance, Jialal and colleagues [4] demonstrated a correlation between PHR and atherosclerosis risk, indicating its potential as a marker for assessing metabolic syndrome and cardiovascular risk. Similarly, a prospective study involving 56,316 patients identified PHR as a promising tool for identifying high-risk individuals among those with CHD and diabetes [5]. Nevertheless further definitive assessments of the association between PHR and cardiovascular risk remain limited. PHR has been proposed as a metabolic indicator for predicting cardiac disease risk, yet definitive evidence supporting its role in identifying future cardiovascular disease risks is lacking and requires further investigation [7].

In this study, significant differences were observed in LDL-C levels among the four groups (Q1 to Q4). Notably, LDL-C levels gradually increased from Q1 to Q3, while a decline was observed in Q4. Previous studies have generally established that a substantial elevation in LDL-C is a primary cause of atherosclerosis. However, numerous observational and experimental studies contradict Bradford-Hill’s causality criteria, failing to establish a clear association between elevated LDL-C levels and the development of atherosclerosis [44]. Furthermore, no controlled, randomized cholesterol-lowering trials in patients with familial hypercholesterolemia (FH) have demonstrated positive outcomes. Additionally, research has suggested that oxidized low density lipoprotein (LDL) promotes platelet activation and arterial thrombosis through scavenger receptors that are constitutively expressed. These receptors transmit lipid-induced stress associated with atherosclerosis to platelets, thereby activating complex signaling pathways that influence thrombus formation and may trigger acute cardiovascular events [45]. This indicates that factors contributing to thrombosis may represent a more significant risk in hyperlipidemia than LDL-C levels alone. Notably, a study has shown that individuals who die prematurely often have elevated levels of lipoprotein(a) [Lp(a)], factor VIII, and/or fibrinogen compared to those with normal life expectancy, while their LDL-C levels do not differ significantly. This suggests that certain FH patients may inherit other genetic risk factors which are more crucial than elevated LDL-C [46].

This study aims to address these gaps by investigating whether PHR can effectively identify individuals at high risk of heart events among middle-aged and elderly adults. Our research, to our knowledge, represents the first attempt to correlate PHR with the incidence of new heart events in this demographic. This investigation could potentially aid in identifying high-risk individuals early, facilitating targeted preventive measures and ultimately extending life expectancy among middle-aged and elderly populations.

## 5. Limitations 

Several limitations are inherent in this study. Firstly, it relies on self-reported data from the study subjects, potentially leading to inaccuracies due to inadequate understanding of their own health conditions, including diabetes, hypertension, and heart disease. Such inaccuracies could affect the reliability of questionnaire responses. Secondly, PHR was assessed only at baseline during the initial follow-up, without capturing dynamic changes over time. Therefore, the relationship between dynamic changes in PHR and the incidence of heart disease remains unknown. Thirdly, this study focused on overall heart disease events without distinguishing between specific types such as heart failure, coronary heart disease, and atrial fibrillation. Future research may benefit from examining these distinctions more closely. Fourthly, as a sampling study, there is a possibility of sampling errors which limit generalizability to the broader population. Lastly, this study did not differentiate between ethnic or national populations in relation to PHR and heart disease events. Future multicenter prospective studies should explore these factors to provide a more comprehensive understanding.

## 6. Conclusions

In conclusion, our study demonstrates a positive correlation between PHR levels and the incidence of heart disease events among middle-aged and elderly individuals in China. Specifically, higher PHR levels correspond to an increased risk of heart disease events, illustrating a linear relationship between PHR and heart disease risk.

## Data Availability

The datasets generated for this study are available on request to the corresponding author.

## Abbreviations

PHR, platelet-to-high-density lipoprotein cholesterol ratio; HDL-C, high-density lipoprotein cholesterol; CHARLS, The China Health and Retirement Longitudinal Study; CVD, cardiovascular disease; CHD, coronary heart disease; NIH, national Institutes of Health; NSF, national Science Foundation; BMI, body mass index; CI, confidence interval; DM, diabetes mellitus; HR, hazard ratios; WBC, white blood cell count; PLT, platelet count; UA, uric acid; BMI, body mass index; TG, triglycerides; TC, total cholesterol; HDL-C, high-density lipoprotein cholesterol; LDL-C, low-density lipoprotein cholesterol; HbA1C, hemoglobin Alc; FBG, fasting blood-glucose; CRP, C-reactive protein; NO, nitric oxide; RCS, restricted cubic splines; ROS, reactive oxygen species; ICBP, international blood pressure consortium; SD, standard deviation; rHDL-C, reconstituted HDL-C; PGI2, prostacyclin; PKB, protein kinase B; MAPK, mitogen-activated protein kinase; AMPK, AMP-activated protein kinase; Lp(a), lipoprotein(a).
